# A Facile *in Situ* and UV Printing Process for Bioinspired Self-Cleaning Surfaces

**DOI:** 10.3390/ma9090738

**Published:** 2016-08-30

**Authors:** Marina A. González Lazo, Ioannis Katrantzis, Sara Dalle Vacche, Feyza Karasu, Yves Leterrier

**Affiliations:** Laboratoire de Technologie des Composites et Polymères (LTC), Ecole Polytechnique Fédérale de Lausanne (EPFL), CH-1015 Lausanne, Switzerland; magonzalezlazo@gmail.com (M.A.G.L.); ioannis.katrantzis@epfl.ch (I.K.); sara.dallevacche@epfl.ch (S.D.V.); feyza.karasukilic@epfl.ch (F.K.)

**Keywords:** self-cleaning, lotus, rose, UV nanoimprint lithography, fluorinated acrylate

## Abstract

A facile *in situ* and UV printing process was demonstrated to create self-cleaning synthetic replica of natural petals and leaves. The process relied on the spontaneous migration of a fluorinated acrylate surfactant (PFUA) within a low-shrinkage acrylated hyperbranched polymer (HBP) and its chemical immobilization at the polymer-air interface. Dilute concentrations of 1 wt. % PFUA saturated the polymer-air interface within 30 min, leading to a ten-fold increase of fluorine concentration at the surface compared with the initial bulk concentration and a water contact angle (WCA) of 108°. A 200 ms flash of UV light was used to chemically crosslink the PFUA at the HBP surface prior to UV printing with a polydimethylsiloxane (PDMS) negative template of red and yellow rose petals and lotus leaves. This flash immobilization hindered the reverse migration of PFUA within the bulk HBP upon contacting the PDMS template, and enabled to produce texturized surfaces with WCA well above 108°. The synthetic red rose petal was hydrophobic (WCA of 125°) and exhibited the adhesive petal effect. It was not superhydrophobic due to insufficient concentration of fluorine at its surface, a result of the very large increase of the surface of the printed texture. The synthetic yellow rose petal was quasi-superhydrophobic (WCA of 143°, roll-off angle of 10°) and its self-cleaning ability was not good also due to lack of fluorine. The synthetic lotus leaf did not accurately replicate the intricate nanotubular crystal structures of the plant. In spite of this, the fluorine concentration at the surface was high enough and the leaf was superhydrophobic (WCA of 151°, roll-off angle below 5°) and also featured self-cleaning properties.

## 1. Introduction

Natural superhydrophobic self-cleaning surfaces such as the famous lotus leaf (*Nelumbo nucifera* [1]) consist of intrinsic hierarchical structures in the nanometer and micrometer ranges based on papillose epidermal cells covered with low surface energy substructures such as epicuticular wax tubules [2,3,4]. The combination of the peculiar sub-micron morphology and the low surface tension favors trapping of small air pockets at the interface with water droplets, which considerably reduces the contact area between the droplet and the surface, resulting in the reduction of contact angle hysteresis, sliding angle, and adhesive force. The self-cleaning effect is the removal of dirt particles by the (rain) water, which forms droplets that do not ‘stick’ on the surface and run away with the dirt. A surface is hydrophobic when its contact angle with water is larger than 90°. Beyond contact angles of 150° with sliding angles (SA) inferior to 10° [5] the surface is considered to be superhydrophobic [6]. Nakajima reported the maximum water contact angle (WCA) on a flat surface to be around 115°, corresponding to a surface energy in the range of 5–7 mJ/m^2^ [7]. Therefore, superhydrophobic surfaces are only attainable by the combination of low surface energy and a surface texturization [8,9]. The hierarchical surface texture of the lotus leaf is well known to be superhydrophobic, with a WCA in the range 162° to 168° [1,10]. For this reason, the hierarchical roughness of the lotus leaves has been widely mimicked or replicated using different technologies and materials [2,11,12,13,14,15] such as polydimethylsiloxane (PDMS) owing to its ability to replicate very small features or structures [16,17,18,19,20]. 

Bioinspired synthetic self-cleaning surfaces often rely on multiple-step and cost-intensive approaches based on conventional surface treatment of textured materials using hydrophobic moieties [21,22,23], or on texturization of inorganic materials using expensive and time-consuming etching processes [24]. A number of approaches have been reported to produce hierarchical dual-roughness structures such as electrodeposition from acidic copper sulfate solution onto flat copper and a patterning technique of coating with a fluorocarbon hydrophobic layer [10] and assembly from colloidal silica-based raspberry-like particles [25]. Record WCA values as high as 179.9° were reached using porous ORMOSIL aerogel thin films [26].

We report an alternative *in situ* approach based on the spontaneous migration of hydrophobic oligomers diluted into polymer precursors to the polymer-air interface and their immobilization through chemical crosslinking with the polymer. For instance, the surface segregation of perfluoropolyether moieties was reported by several authors [27,28,29,30]. Fluorine increases the molecular cross section of the base polymer and thus makes it more hydrophobic [31]. Then, when polymerization takes place, the fluorinated moieties are chemically crosslinked with the polymer network and the surface is permanently modified [27]. This migration effect was thus expected to replace the additional hydrophobization step commonly used to create self-cleaning surfaces. 

The paper details the original combination of this spontaneous migration with a cost-effective, fast and low-pressure UV nano-imprint lithography (UVNIL) process [16,17]. Lotus leaves and rose petals were selected since these plants exhibit contrasted wetting properties, the latter being characterized by an adhesive behavior with large SA values [32,33,34]. An acrylated hyperbranched polymer (HBP) was used to benefit from its low shrinkage behavior and resulting very high replication fidelity in nano-printing processes [35,36,37,38] and a glass transition temperature well above room temperature. Transparent PDMS molds texturized with negative replica of the plant surfaces were used. Our single-step *in situ* process turned out to be challenging due to the reverse migration of the hydrophobic oligomers back into the bulk polymer when put in contact with the PDMS mold surface. The solution was found by applying a flash of UV light to immobilize the oligomers through superficial crosslinking, as detailed in the following. We first studied the segregation of a fluorinated oligomer at the polymer-air interface on flat HBP coatings. In a second step we replicated the hierarchical nanostructures of the plant surfaces using the PDMS casting technique. In a third step, UVNIL was performed using the PDMS templates and the HBP mixed with the fluorinated oligomer, without and with a UV flash prior to molding. The quality of the replicas was characterized using electron microscopy. The hydrophobic character of both flat and texturized surfaces, with or without fluorine was investigated using WCA measurements. The self-cleaning ability of the synthetic plant surfaces was finally investigated and demonstrated for the synthetic lotus leaves exposed to a flash of UV light prior to UVNIL.

## 2. Materials and Methods

**Figure 1:**
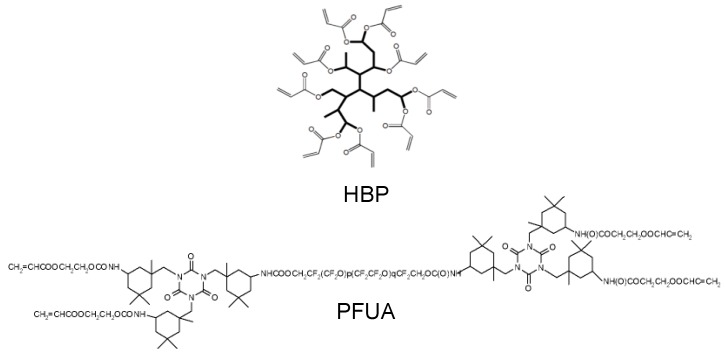
Chemical structure of HBP and PFUA.

A hyperbranched polyester acrylate oligomer (HBP CN2302, Sartomer, Verneuil-en-Halatte, France, Figure 1) with a functionality of 16, a density of 1.13 g·cm^−3^, a newtonian viscosity of 0.2 Pa·s at 25 °C and a glass transition temperature in the cured state of 74 °C was selected owing to its low viscosity and low shrinkage, which warrant improved replication fidelity [35,38]. The fluorinated surfactant was a tetrafunctional perfluoropolyether-urethane acrylate (PFUA, Fluorolink AD1700, Solvay Specialty Polymers, Bollate, Italy, Figure 1), with molecular weight equal to 4000 Da and fluorine content equal to 24 wt. %. PFUA was diluted at a concentration of 70 wt. % in a mixture of ethylacetate (14 wt. %) and *n*-butylacetate (16 wt. %). The photo-initiator was 2,4,6 trimethylbenzoyldiphenyl phosphine oxide (Esacure TPO, IGM Resins, Gerenzano, Italy). The TPO photo-initiator was first dissolved in the HBP at 75 °C and at a concentration of 6 wt. % and stirred for 15 min. In a second step, different concentrations of PFUA ranging from 0.5 to 5 wt. % were added. The mixtures were then stirred for 15 min at 40 °C. The solvents were evaporated during this process. The main issues to prepare the HBP-PFUA blends were the high viscosity of the PFUA and the polarity difference between the two constituents resulting in inhomogeneous solutions beyond 1 wt. % of PFUA as previously reported [39]. In all cases, the PFUA had a tendency to phase separate from HBP with time, so it was essential to remix the solutions prior to any measurement and further processing. The thermal and light stability of the cured HBP-PFUA blends were not investigated. However, the HBP was found to be stable under oxygen up to approximately 400 °C [38], and fluorine substitution is known to improve the thermal stability of polyacrylates [40], which warrants high thermal endurance of the synthesized coatings. Moreover, the addition of suitable stabilizers and perfluorinated moieties was also reported to further improve the long-term weathering [41], and yellowing resistance of similar materials [42].

Around 200 μm thick coatings were prepared on glass slides. To study the influence of air exposition time on the migration of the PFUA to the polymer-air interface [28,39], the samples were polymerized right after the coating step, or after 30 min, 2 h and 24 h of air exposure in a dark cabinet. A 200 W high-pressure mercury UV lamp (OmniCure 2000, Exfo, Quebec, QC, Canada) was used to cure the materials. The light intensity between 230 and 410 nm was measured using a calibrated radiometer (Silver Line, CON-TROL-CURE, Brachttal, Germany). All samples were irradiated during 3 min under a UV intensity of 75 mW·cm^−2^. 

**Figure 2:**
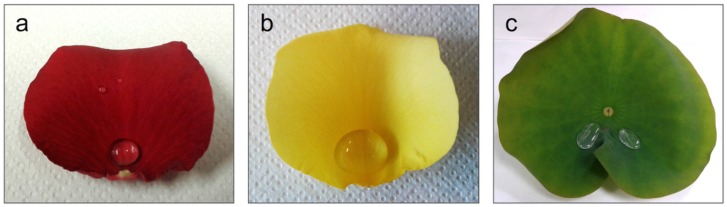
Photographs of (**a**) red rose petal; (**b**) yellow rose petal; and (**c**) lotus leaf with water droplets on them.

Three different plant surfaces were selected, namely leaves from *Nelumbo Lutea* (lotus) and petals from *Rosa rosa* (red rose) and *Rosa hemisphaerica* (yellow rose). Figure 2 shows the three surfaces with water droplets on the top. The plant surfaces were replicated into the polymer materials using an intermediate negative PDMS master (Sylgard 184, Dow Corning, Midland, MI, USA). This soft, vacuum-free and ambient molding technique preserved the delicate biological surfaces from damage, and enabled to accurately reproduce their nanometer scale features. Square samples (2 cm × 2 cm) cut from the lotus leaf and from the rose petals were fixed onto Petri dishes. PDMS mixed with hardener (10:1 ratio) was poured onto the samples and air bubbles were removed under a reduced pressure for 20 min. The PDMS was subsequently cured at room temperature for 48 h. The resulting PDMS template (which is the negative of the plant surfaces) was used to imprint HBP and HBP with PFUA with a UVNIL tool equipped with independent control of UV exposure and pressure as detailed in a previous work [43]. Samples were irradiated for 3 min under a pressure of 3 bars and a UV intensity of 75 mW·cm^–2^.

Water contact angles were measured using a contact angle meter (EasyDrop, Krüss GmbH, Hamburg, Germany) at room temperature using deionized water, enabling controlled dispensing of 9.3 µL water drops. This volume corresponds to a spherical drop diameter of approximately 2.6 mm, much larger than the micrometer features of the investigated surfaces and small enough to reach a Bond number as low as approximately 2 × 10^−3^ so that gravity effects were negligible. The sessile drop technique was selected and the advancing contact angle was measured. The WCA of three to five droplets was measured on each sample and the values were averaged.

X-Ray photoelectron spectroscopy (XPS) data of the surface of fluorinated HBP coatings were collected by Axis Ultra (Kratos analytical, Manchester, UK) under ultra-high vacuum condition (<10^−8^ Torr), using a monochromatic Al K_a_ X-ray source (1486.6 eV). The source power was maintained at 150 W (10 mA, 15 kV). The emitted photoelectrons were sampled from a square area of 750 µm × 350 µm. Gold (Au 4*f*_7/2_) and copper (Cu 2*p*_3/2_) lines at 84.0 eV and 932.6 eV, respectively, were used for calibration, and the adventitious carbon 1s peak at 285 eV was also used as an internal standard to compensate for any charging effects. 

The topography of the replicated surfaces was examined using a scanning electron microscope (SEM, FEI XL30-SFEG, FEI, Hillsboro, OR, USA) operated in ultra-high resolution mode, using an acceleration voltage of 10 kV. The samples were coated with a carbon layer to avoid charging effects.

## 3. Results and Discussion

### 3.1. Migration of Fluorinated Oligomers Towards HBP-Air Interface

**Figure 3:**
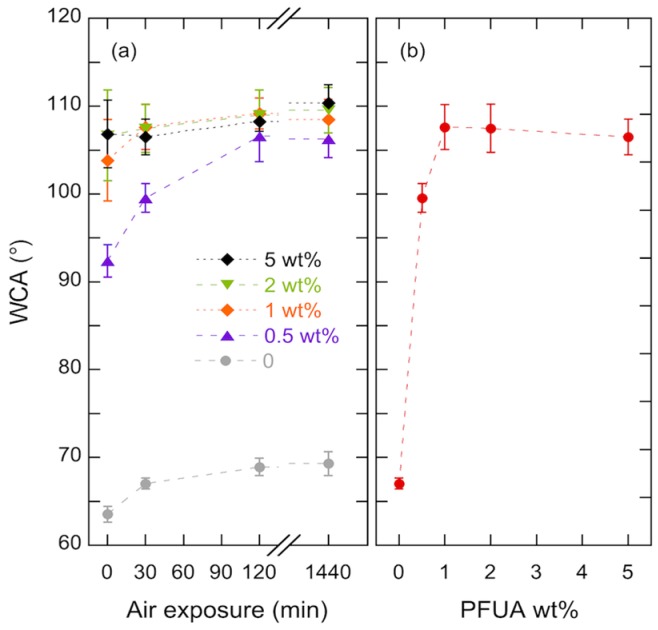
Water contact angle vs. (**a**) air exposition time for pure HBP and fluorinated flat surfaces for different concentrations of PFUA as indicated; and (**b**) PFUA concentration for HBP coatings polymerized after 30 min of air exposure.

Figure 3 shows the WCA for pure HBP coatings and HBP coatings with different concentrations of PFUA that were polymerized after 0 min, 30 min, 2 h or 24 h of air exposure. The pure HBP polymerized right after the coating step had a WCA of 63.5°. When the HBP was exposed to air for 24 h prior to polymerization, the WCA increased to 69.3°, a likely result of structural reorganization of hydrophobic and hydrophilic segments of the molecules in order to become better segregated within the coating [44]. With 0.5 wt. % of PFUA the WCA immediately reached a value of 92.4° and further increased to 99.6° for 30 min of air exposition prior to polymerization and to 106.3° after 24 h. With concentrations of 1 wt. % of PFUA, the WCA immediately reached a value of 104°, and saturated to a value of 109° after 2 h. The hydrophobization behavior was the same within experimental scatter for all investigated concentrations of PFUA beyond 1 wt. %. The longer the sample was exposed to air, the higher was the contact angle, as previously reported by Bongiovanni et al. [45]. This result demonstrates a fluorine migration across the sample towards the polymer-air interface, where it accumulates and saturates. The reason is that the composition of coatings at the interface with polar substrates such as glass is different from their composition on the air side, as a consequence of the interfacial energy minimization [27,46].

The saturation effect is exemplified in Figure 3b, where the contact angle after 30 min of air exposure is shown as a function of the concentration of PFUA. A steep increase in hydrophobicity with 40° increase in WCA is evident with the addition of 1 wt. % of PFUA. Further addition of PFUA had no more effect on the WCA, which remained equal to approximately 108°. This result is consistent with previous studies, which report comparable contact angle values with a similar fluorinated surfactant [28,29,30]. Notice that significant experimental scatter arose from two different effects depending on the concentration of PFUA in the sample, resulting in some degree of surface heterogeneity. For concentrations below 1 wt. %, there was not enough PFUA to saturate the whole sample surface, whereas, for concentrations above 1 wt. % samples were visually inhomogeneous due to segregation of the highly viscous PFUA. In both cases, heterogeneity in surface composition was on the same size range as the water droplets. 

**Figure 4:**
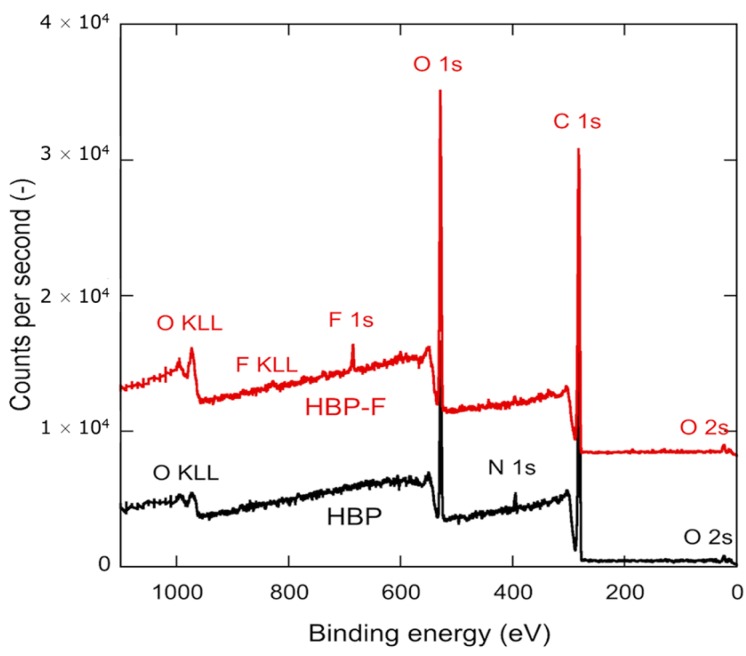
XPS analyses of non-fluorinated HBP (HBP, **black** spectrum) and fluorinated HBP (HBP + 1.2 wt. % of PFUA and 30 min of air exposure, HBP-F, **red** spectrum) surfaces.

Figure 4 compares the chemical composition within a 5–10 nm thick superficial layer of the HBP and HBP + 1.2 wt. % of PFUA (i.e., 0.29 wt. % of fluorine according to the concentration of fluorine in PFUA of 24 wt. %) exposed to air for 30 min prior to polymerization, as determined through XPS. The presence of fluorine was detected only in the case of the fluorinated sample.

The corresponding surface composition of the two materials is detailed in Table 1. The analysis confirmed the absence of fluorine at the surface of the HBP. It also revealed a 2.5% mass concentration of fluorine at the surface of the HBP-PFUA sample, i.e., approximately 10 times more than the initial concentration of fluorine in the mixture, a clear demonstration of the migration of PFUA molecules to the surface. The saturated concentration of PFUA at the polymer surface would thus be 10 times the threshold concentration of 1 wt. % shown in Figure 3b, i.e., around 10 wt. %.

### 3.2. Replication of the Rose Petals and Lotus Leaf into HBP-F

**Figure 5:**
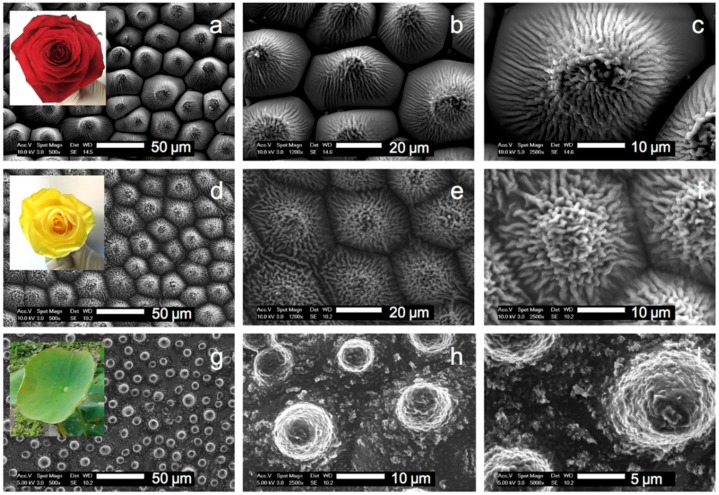
Electron micrographs at three different magnifications of HBP replica of a red rose petal (**a**–**c**); a yellow rose petal (**d**–**f**) and a lotus leaf (**g**–**i**). The insets show the pictures of the three plants used in this work.

Figure 5 shows the synthetic HBP replica of the three plant surfaces. The morphologies perfectly mimic the surfaces of the original plants. In particular, the closely packed array of micropapillae and nanofolds on each papilla top is apparent on the replicated petal structures [32]. The characteristic features of papillose epidermal cells of the lotus leaf, which are smaller and not as densely packed compared with the rose petal structures are also evident [16,17], although one may notice the absence of part of the well-defined epicuticular wax nanotubules. Different surface densities of micropapillae on these three surfaces are also evident, being the highest for the lotus (~3500 mm^−2^), followed by the yellow rose (~1600 mm^−2^) and the red rose (~1000 mm^−2^).

### 3.3. Dilution of the Fluorinated Oligomer on the Texturized Surface and Reverse Migration

The WCA of flat and texturized surfaces are reported in Table 2. Clearly, the WCA increased significantly upon texturization for the three investigated plants, from 69° for the flat HBP to 120° for the red rose HBP, 126° for the yellow rose HBP and 133° for the lotus HBP. Texturization changed the hydrophilic HBP (WCA < 90°) into a hydrophobic surface (WCA > 90°) more efficiently than through a reduction of the surface energy with the addition of PFUA (WCA of 108°, see also [7]). The impact of tailored hierarchical structures at micron and nanometer scales on large WCA increases is indeed significant [47], unless the texturized material is hydrophilic, such as alumina [48]. In this latter case, the addition of a hydrophobic surface treatment is essential to achieve the requested WCA increase beyond 150°. Interestingly, an opposite hydrophobic ranking was found for the negative PDMS replica, the negative red rose surface being the most hydrophobic one. However, and surprisingly, the addition of 5 wt. % of PFUA, without or with migration did not significantly change the WCA of the synthetic red rose and lotus surfaces, which was unexpected. WCA was increased by approximately 10° for the synthetic yellow rose, but 2 h of air exposure did not have much influence, in striking contrast with the results obtained on flat surfaces depicted in Figure 3. Two possible effects were considered to explain the observed puzzling results.

The first effect was a dilution of the superficial concentration of fluorine, as a result of the large increase of roughness of the texturized surfaces compared with the flat surfaces. The corresponding surface increase (also termed roughness factor) is 3.2 for lotus leaves [49] and as high as 9.2 for rose petals with respective contributions from the nanofolds and micropapillae of 7.87 and 1.32 [50]. In this latter work, the authors did not specify the type of roses, which were presumably red roses according to the electron micrograph shown in [50]. The roughness factor is likely to be even higher for the yellow rose petal with higher areal density of papillose cells with higher number of nanofolds as seen in Figure 5. The surface concentration of fluorine decreases in proportion with the roughness factor. The fluorine surface concentration of 2.5 wt. % (XPS data, Table 1) was thus diluted by a factor of 3.2 when the flat surface is imprinted with a lotus texture, and by 9 times or more in the case of rose petals. The resulting concentrations of fluorine at the surface would then be insufficient and the WCA would not increase. Additional tests, with increasing initial concentration of PFUA to compensate for this dilution effect and increasing air exposure to maximize surface segregation were carried out. These attempts were not successful: an initial 5 wt. % of PFUA and 2 h of air exposure led to no or marginal increases of WCA as reported in Table 2 for the three plant textures.

**Figure 6:**
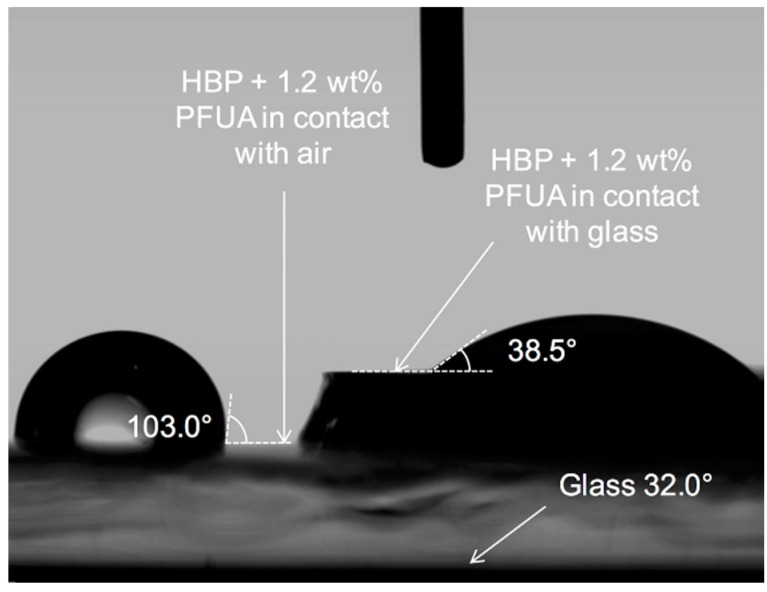
Morphology of water droplets on HBP with surface segregated PFUA in contact with air (**left** droplet) and with glass (**right** droplet) during UV curing.

The second effect was the reverse migration of the PFUA into the bulk of the polymer coating upon contact with the PDMS mold surface. The following set of experiments confirmed that this was indeed the case. In these experiments, HBP coatings with 1.2 wt. % of PFUA were produced on a glass slide, exposed to air for 30 min to enable migration, and then coated with either a 1 mm thick glass slide or a 5 mm thick PDMS layer placed on the top, fluorine-rich surface of the coating and finally UV cured. The UV intensity was adjusted to compensate for the absorption through the top glass slide. The top glass slide or PDMS layer was removed after complete polymerization prior to contact angle measurement. Detachment of the cured polymer from these two surfaces turned out to be easy. In the case of the glass slide a coating area with reduced thickness was left in contact with the air in-between the two glass substrates for comparison. Figure 6 shows 2 water droplets, one on the coating surface in contact with air during UV cure, and the other in contact with glass during UV cure.

The droplet on the left exhibits a WCA of 103°, similar to the experiments shown in Figure 3 while the droplet on the right presents a WCA of only 38.5°. The WCA of the bare glass substrate was measured for comparison and was found to be equal to 32°. This value presumably indicates the presence of residual contamination since the WCA on hydrophilic silanol surface should be 0°. An exposition of 30 min to air was enough to accumulate enough PFUA on the coating-air interface to obtain a hydrophobic behavior. However, the WCA value of 38.5° implied that the presence of a glass substrate disrupted the migration of the PFUA in the coating and caused a steep decrease in hydrophobicity. The same experience was carried out using a PDMS flat layer (acting as non-polar material) instead of glass. After removal of the PDMS top layer, WCA of 71.6° and 97.5° were found for the HBP and HBP + 1 wt. % of PFUA and 2 h of air exposure, respectively. This result further demonstrates that the type of material in contact with the polymer prior to UV curing has indeed an influence on the surface energy of the cured polymer. Curing the HBP without PFUA in contact with glass rather than with air led to a WCA of 37.3°. A tentative explanation of this behavior is found when considering the interfacial interactions between glass and the HBP. The surface of glass, prior to the application of the HBP coating, is composed of silanol (Si–OH) groups, with density depending on glass composition and ambient relative humidity [51]. The acrylate groups of the HBP form H–bonds with the silanol groups [37], which immobilize the acrylate and may prevent further reaction. Upon release from the glass after polymerization, these rather weak bonds break and the unreacted acrylates would explain the easy separation of the cured HBP from glass, and its increased hydrophilicity on the glass side. 

### 3.4. UV Flash Immobilization of Fluorinated Oligomer at the HBP-Air Interface

In order to suppress the reverse migration effect of the PFUA upon contacting the PDMS mold, the HBP-PFUA mixture was exposed to air for 30 min to enable migration and then irradiated under UV light for a short time to crosslink the superficial layers of the coating. The duration of this UV flash was short enough to prevent gelation of the bulk coating [52], which would compromise the subsequent low-pressure UVNIL process. Experiments were performed with HBP coatings containing 5 wt. % PFUA, first exposed to air for 2 h to maximize surface concentration of fluorine. The coatings were then irradiated under UV light with an intensity of 75 mW/cm^2^ for durations ranging from 0.2 to 2 s. The flashed coatings were loaded into the UVNIL tool and their surface was printed with the three types of plant textures, using the same illumination time and pressure as for the previous experiments. It turned out that the replication fidelity was as good as that obtained without UV flash for the two rose petal surfaces with densely packed micropapillae, but not for the lotus surface. In that case, parts of the surface were not properly printed, leaving flat areas between papillose cells. This was solved by maintaining the polymer sample under a pressure of 4 bars for 5 min for viscous flow to occur, prior to UV exposure. 

**Figure 7:**
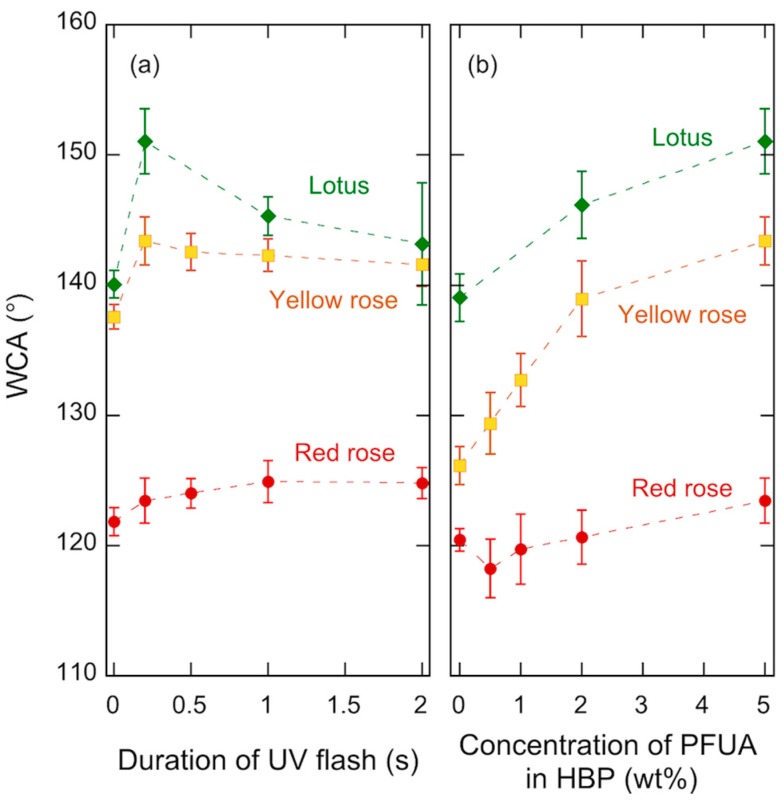
WCA of synthetic plant surfaces based on HBP + PFUA with 2 h of air exposure vs. (**a**) duration of UV flash prior to UVNIL for a 5 wt. % concentration of PFUA and (**b**) concentration of PFUA in HBP for a 0.2 s UV flash prior to UVNIL.

Figure 7 shows the WCA of the synthetic replica of the three plants vs. duration of the UV flash, and vs. concentration of PFUA for a 200 ms UV flash. The highest WCA was on a lotus leaf replica, with a value of 151°. This value was significantly lower than that of the natural plant and is likely due to the absence of nanotubules on the polymer replica, which has been shown to reduce the superhydrophobic behavior of such surfaces [13]. The highest WCA value on the red rose surface was 123°, much lower than the value of 153° reported for the natural red rose petal [53]. This is a probable result of the large decrease of fluorine concentration at the surface resulting from the large increase of the roughness upon printing as detailed in the following.

As shown in Figure 7a, a 200 ms UV flash was sufficient to increase the WCA, by approximately 5° for the yellow rose and by more than 10° for the lotus. For these two plants longer flashes led a decrease of the WCA and an increased experimental scatter, which was particularly pronounced in case of lotus. Such reduction of the WCA was due to degraded replication fidelity, a result of the increased viscosity of the polymer, and points out the importance of careful process control in such UV printing processes. An increase of the WCA on the synthetic red rose surface also occurred with a flash of UV light, but the increase was less than 3°, and longer flashes were needed. It is moreover evident from Figure 7b that the WCA increased with increasing concentration of PFUA for both lotus and yellow rose, the trend being not clear for the red rose. This suggests that the dilution effect upon surface texturization was present and was quite significant for concentrations below 2 wt. %, which is in fact consistent with the threshold concentration of 1 wt. % (Figure 3) considering a roughness factor close to 3 for lotus. As discussed previously, the much higher roughness factor of the yellow rose petals would explain the systematic lower WCA on the yellow rose compared with that on the lotus.

The considerably different wetting behavior of the investigated surfaces reflect the complex interplay between hierarchical surface morphologies, surface energy and trapping of air pockets at the liquid-solid interface, a critical factor for superhydrophobicity. It is generally recognized that micropapillae with higher aspect ratio and higher areal density present increased WCA and reduced SA [34]. In the present case, the red rose petals had the lowest areal density and the concentration of PFUA at their surface was probably below the saturation value of 10 wt. % due to a large dilution effect. In addition, the nanofolds on the red rose papillose cells form directional grooves, which favors anchoring of water droplets [50,53]. The density of micropapillae was higher for the yellow rose, and the nanofold morphology resembled the nanotubular structure of the lotus wax crystals. This presumably explains the large increase of hydrophobicity of this type of rose petal compared with the red rose petal. However, the roughness ratio was probably too high, and the resulting surface concentration of PFUA upon texturization was again insufficient for superhydrophobicity. Finally, the synthetic lotus surface combined a high density of micropapillae and a high enough concentration of fluorine due to a relatively small roughness ratio and, in spite of incomplete replication of the nanotubules, it was superhydrophobic.

### 3.5. Self-cleaning Synthetic Plant Surfaces

**Figure 8:**
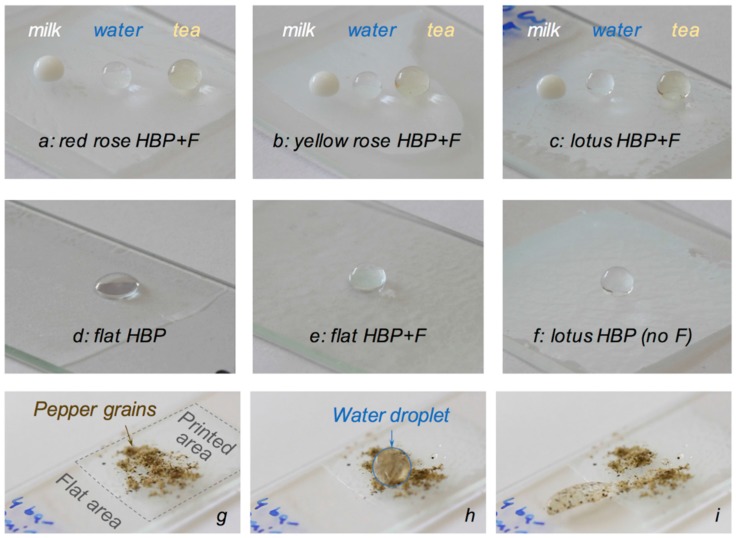
Behavior of milk, water and tea on the surfaces of flat and printed HBP and fluorinated HBP (HBP + F) coatings on glass slides (liquid droplets on HBP + 5 wt. % PFUA with 2 h air exposure and 0.2 s UV flash replica of (**a**) red rose; (**b**) yellow rose and (**c**) lotus; water droplets on (**d**) flat HBP; (**e**) flat HBP + 5 wt. % PFUA with 24 h air exposure; (**f**) lotus replica of pure HBP) and demonstration of the self-cleaning properties of a lotus replica (HBP + 5 wt. % PFUA with 2 h air exposure and 0.2 s UV flash) using water and pepper grains; (**g**) before cleaning with water; (**h**) with a water droplet (highlighted with a blue circle) running on the pepper contaminated surface and (**i**) after cleaning, with water droplet loaded with pepper particles and immobilized on the non-printed, flat area, leaving a clean trace on the printed surface.

The self-cleaning ability of the three plant replicas was tested against water, milk and tea. Figure 8 shows droplets of these three liquids on the three synthetic plant surfaces found to exhibit the highest WCA (i.e., with an initial bulk concentration of PFUA of 5 wt. %, an air exposure of 2 h and a UV flash of 200 ms, see Figure 7), and a sequence of three pictures which exemplify the effective cleaning with water of a synthetic lotus surface contaminated by pepper grains. Removal of hydrophobic contaminants such as pepper grains with water droplets is more challenging than model hydrophilic contaminants such as MnO powder [54] and SiC powder [55], because these grains would adhere well to the hydrophobic surface. In that case, the weak adhesive forces between the hydrophobic particles and water overcome the adhesive forces between the particles and the texturized surface, because these are very low due to very small contact area between the particles and the top of the textures, and the particles are captured by the droplets. Water droplets on control surfaces (flat HBP, flat fluorinated HBP and HBP lotus replica without fluorine) are also shown. The latter three surfaces were not self-cleaning. Interestingly, neither were the two fluorinated lotus replica, for which no air exposure nor UV flash were used. 

The sliding angle of water on the synthetic red rose surface was measured and found to be equal to approximately 60°, and several water droplets remained attached to the surface when the sample was turned upside-down. This confirmed the adhesive, Cassie impregnating wetting state also termed ‘petal-effect’ of this surface [32,33,34], which did not show any self-cleaning behavior with the selected liquids. The value of the SA of water on the synthetic yellow rose was approximately 10°, at the limit for superhydrophobic surfaces, as previously noticed from its WCA of 143°. Water and milk droplets rolled on the synthetic yellow rose petal. However, its self-cleaning ability was not very good, which might be improved with increasing concentration levels of PFUA to overcome the strong dilution effect discussed above. The SA of water on the synthetic lotus surface was equal to 5° (at an angle of 3°, most but not all water droplets did roll on the surface), thus confirming its superhydrophobic character. This surface was self-cleaning with water, but not with milk.

## 4. Conclusions

Bioinspired self-cleaning surfaces based on synthetic replica of plant surfaces were developed using an acrylated hyperbranched polymer (HBP) and a fluorinated acrylate surfactant (PFUA). A PDMS negative template of red and yellow rose petals and lotus leaves and a UVNIL process were used. The degree of hydrophobicity of the synthetic replica was investigated with attention paid to the concentration of PFUA and segregation of PFUA at the polymer-air interface. The analysis of the experimental results led to the following conclusions.

Dilute concentrations of 1 wt. % PFUA in 200 µm thick HBP coatings migrated to the polymer-air interface within 30 min, leading to a ten-fold increase of the amount of fluorine at the surface and a mass concentration equal to 2.5%. This migration process was found to be reversible when the polymer-air interface was changed to polymer-glass and polymer-PDMS interfaces, but became irreversible through photo-crosslinking of the superficial layers with a 200 ms flash of UV light. The hydrophilic HBP surface (WCA of 69°) became hydrophobic (WCA of 108°) with an initial concentration as low as 1 wt. % of PFUA followed by a 30 min air exposure. Increasing the concentration of PFUA beyond 1 wt. % did not further increase the WCA.

The rapid, low-pressure UVNIL process based on the low-shrinkage HBP precursor enabled a very high replication fidelity of the surfaces of the original plants, with well resolved arrays of papillose cells and their sub-micron cuticular features. Using the spontaneous migration and UV flash crosslinking processes, the surface textures of the three plants were successfully printed into the HBP coatings with fluorinated surface. However, for the rose petals based on HBP with a bulk concentration of 5 wt. % of PFUA, the surface concentration of fluorine was below the saturation value for superhydrophobicity, due to a dilution effect upon printing the petal textures with very high roughness. The synthetic red rose petal was hydrophobic (WCA of 125°) and adhesive and the synthetic yellow rose petal was quasi-superhydrophobic (WCA of 143°, roll-off angle of 10°). The surface concentration of fluorine on the synthetic lotus leaf reached the saturation value and the leaf was superhydrophobic (WCA of 151°, roll-off angle of 5°) and featured self-cleaning properties with water.

## Figures and Tables

**Table 1:** XPS analysis of the surface of HBP and HBP + 1.2 wt. % of PFUA with 30 min of air exposure.

Sample	Peak	Position Beam Energy (eV)	Full Width at Half Maximum (eV)	Raw Area (CPS)	Relative Sensitivity Factor	Atomic Mass	Atomic Conc. * (%)	Mass Conc. * (%)
HBP	F 1s	687.3	0.000	0.0	1.000	18.998	0.00	0.00
	O 1s	528.6	2.363	26,010.7	0.780	15.999	16.60	20.95
	C 1s	280.8	2.366	42,133.2	0.278	12.011	83.40	79.05
HBP-PFUA	F 1s	685.3	2.363	3317.5	1.000	18.998	1.70	2.46
	O 1s	528.7	2.431	35,280.0	0.780	15.999	24.19	29.56
	C 1s	281.9	2.773	34,850.6	0.278	12.011	74.11	67.98

* Conc. = concentration.

**Table 2:** Influences of air exposure prior to UV polymerization and concentration of PFUA on WCA of flat and texturized HBP and fluorinated HBP coatings. The WCA of flat HBP and fluorinated HBP polymerized in contact with glass and PDMS, of glass and of PDMS, and of PDMS negative replica of the plants are also reported.

Surface	Material	Air Exposure (min)	Template Material	WCA (°)
Flat	HBP	–	–	68.7 ± 1.2
	HBP + 1 wt. % PFUA	30	–	107.6 ± 2.6
	Glass *	–	–	32.0
	PDMS *	–	–	108.7
	HBP *	–	Glass	37.3
	HBP	–	PDMS	71.6 ± 1.7
	HBP + 1 wt. % PFUA *	0	Glass	38.5
	HBP + 1 wt. % PFUA	120	PDMS	97.5 ± 1.1
Red rose petal texture	HBP	–	PDMS	120.4 ± 0.9
	HBP + 5 wt. % PFUA	0	PDMS	117.8 ± 2.3
	HBP + 5 wt. % PFUA	120	PDMS	121.9 ± 1.1
Yellow rose petal texture	HBP	–	PDMS	126.2 ± 1.5
	HBP + 5 wt. % PFUA	0	PDMS	136.5 ± 2.0
	HBP + 5 wt. % PFUA	120	PDMS	137.6 ± 0.9
Lotus leaf texture	HBP	–	PDMS	139.1 ± 1.8
	HBP + 5 wt. % PFUA	0	PDMS	141.7 ± 2.0
	HBP + 5 wt. % PFUA	120	PDMS	140.1 ± 1.1
Negative red rose	PDMS	–	Red rose	134.3 ± 5.3
Negative yellow rose	PDMS	–	Yellow rose	131.3 ± 4.1
Negative lotus	PDMS	–	Lotus	117.3 ± 2.6

* Only one measurement available.
